# Supplementary material for: Predicting lymphatic filariasis elimination in data-limited settings: A reconstructive computational framework for combining data generation and model discovery
Source: PLoS Comput Biol. 2020 Jul 21;16(7):e1007506. doi: 10.1371/journal.pcbi.1007506 (PMC7394457; doi:10.1371/journal.pcbi.1007506)
Supplement: S1 File — (DOCX) [file pcbi.1007506.s001.docx]

# Supporting Information:

# Predicting lymphatic filariasis elimination in data-limited settings: a reconstructive computational framework for combining data generation and model discovery

Morgan E. Smith, Emily Griswold, Brajendra K. Singh, Emmanuel Miri, Abel Eigege, Solomon Adelamo, John Umaru, Kenrick Nwodu, Yohanna Sambo, Jonathan Kadimbo, Jacob Danboyi, Frank O. Richards, Edwin Michael

## Survey methodology and data aggregation

#### Nigerian sites

The Nigerian analysis drew from papers published by TCC staff, reports and presentations on the program, unpublished manuscripts, monitoring data, and treatment registers. Data were triangulated to improve accuracy. After compiling data available at TCC headquarters in Atlanta, TCC Nigeria staff were brought in to supplement and elucidate the dossiers.

Indicators of human cases of lymphatic filariasis focused on measuring mf and CFA. The sampling method was typically a convenience sample of persons residing in the respective village, except during the baseline (1999-2000) where a random selection of 50 adults was taken. Mf were typically assessed using thick blood smears, while antigenemia was evaluated using ICT kit cards. Epidemiology surveys typically occurred annually beginning three years after baseline. Mf was first assessed in 2002, and ICT tests were done at the beginning of TCC’s involvement and again annually from 2003 to 2011.

Treatments were administered and managed by community-based drug distributors (CDDs). CDDs tracked eligibility and treatment information, forwarding it to local supervisors, who then sent it to TCC staff. Annual MDAs occurred after any epidemiological surveys had finished for that year. The program made efforts to ensure drugs were delivered at a time of year that would not conflict with busy planting or harvesting seasons, although this was not always successful. The last IVM+ALB treatments were given in 2012. ALB distribution continued to certain groups for treatment of soil-transmitted helminths, though this was not considered in the modeling exercises. MDA with IVM continued after 2012 for onchocerciasis, and this was considered in the modeling exercises.

Data were systematically entered into an Access database, and then exported to Excel for manipulation and triangulation. The goal was to obtain one single data point per village per year. Field staff reviewed the reports and then gathered additional files, which were then merged with existing data. Published data and cleaned outputs from unpublished surveys took precedence over regular reports from the field.

#### Global sites

The details of the surveys conducted in Tanzania, PNG, and Egypt are given elsewhere and all data used in this study come from publications [1-5].

Table A: Timeline of key interventions, milestones, and surveys in Seri, Nigeria.

| **Year** | **Intervention/Milestone** |
| --- | --- |
| 1995 | Ivermectin treatment for onchocerciasis begins |
| 2000 | Baseline survey in Seri; addition of albendazole to TCC LF program |
| 2001 | Combination IVM+ALB treatment begins in Seri |
| 2004 | ITNs distributed to vulnerable groups |
| 2009 | First distribution of LLINs |
| 2010 – 2011 | Second distribution of LLINs |
| 2012 | TAS; decision to stop LF treatment in Kanke LGA; IVM continues for onchocerciasis |
| 2017 | Final TAS in Plateau state, LF eliminated as a public health problem |

Table B. Baseline and monitoring epidemiological survey data and intervention details for Dokan Tofa and Piapung, Nigeria.

| **Village** | Dokan Tofa | | | Piapung | | |
| --- | --- | --- | --- | --- | --- | --- |
| **Year** | **% mf prevalence (no. sampled)** | **% CFA prevalence (no. sampled)** | **MDA Coverage (% population) (regimen)^2^** | **% mf prevalence (no. sampled)** | **% CFA prevalence (no. sampled)** | **MDA Coverage (% population) (regimen)^2^** |
| 2002 | 5.0 (419)^1^ | 23.2 (419)^1^ | N/A | 9.9 (403)^1^ | 30.3 (403)^1^ | N/A |
| 2003 | - | - | 74.9 (I+A) | - | - | 70.2 (I+A) |
| 2004 | - | - | 76.7 (I+A) | - | - | 72.0 (I+A) |
| 2005 | 3.0 (236) | - | 67.4 (I+A) | 4.3 (256) | 24.0 (192) | 78.0 (I+A) |
| 2006 | 0.0 (132) | - | 77.6 (I+A) | 3.8 (160) | - | 78.5 (I+A) |
| 2007 | 1.3 (151) | 14.4 (277) | 77.1 (I+A) | 9.6 (187) | 19.6 (312) | 80.1 (I+A) |
| 2008 | 0.0 (158) | 8.7 (158) | 78.3 (I+A) | - | 14.5 (62) | 79.2 (I+A) |
| 2009 | 0.4 (223) | 3.0 (223) | 78.2 (I+A) | 2.1 (291) | 9.3 (291) | 78.9 (I+A) |
| 2010 | 0.0 (119) | 2.5 (119) | - | - | - | - |
| 2011 | 0.5 (206) | 3.4 (206) | - | 1.1 (90) | 2.2 (90) | - |
| 2012 | - | - | - | - | - | - |

N/A not applicable; - not available

^1^ Baseline survey with age-stratified infection data available

^2^ I: ivermectin, A: albendazole

Table C. Baseline and monitoring epidemiological survey data and intervention details for three LF endemic sites from various regions.

| **Village** | Kirare, Tanzania | | | Giza, Egypt | | | Usino Bundi, PNG | | |
| --- | --- | --- | --- | --- | --- | --- | --- | --- | --- |
| **Year** | **% mf prevalence (no. sampled)** | **% CFA prevalence (no. sampled)** | **MDA Coverage (% population) (regimen)^3^** | **% mf prevalence (no. sampled)** | **% CFA prevalence (no. sampled)** | **MDA Coverage (% population) (regimen)^3^** | **% mf prevalence (no. sampled)** | **% CFA prevalence (no. sampled)** | **MDA Coverage (% population) (regimen)^3^** |
| 1 | 26.1 (471)^1^ | 53.3 (90)^2^ | 64.0 (I+A) | 11.5 (1067)^2^ | 19.0 (1067)^2^ | 86.7 (D+A) | 14.0 (757)^1^ | 47.5 (558)^1^ | 68.4 (D+A) |
| 2 | 20.8 (461) | 53.0 (66) | 76.0 (I+A) | 4.5 (1012) | 16.0 (1012) | 95.5 (D+A) | 8.3 (696) | 35.1 (692) | 76.4 (D+A) |
| 3 | 15.8 (438) | 51.4 (72) | 69.6 (I+A) | 2.7 (1026) | 10.5 (1026) | 90.1 (D+A) | 3.4 (714) | 25.2 (695) | 73.9 (D+A) |
| 4 | 10.0 (351) | 52.5 (61) | N/A | 1.3 (1010) | 5.2 (1010) | 88.8 (D+A) | 1.3 (529) | 17.1 (543) | N/A |
| 5 | 12.9 (302) | 44.9 (49) | 77.3 (I+A) | 0.4 (1116) | 2.8 (1116) | 90.3^5^ (D+A) | N/A | N/A | N/A |
| 6 | 5.0 (259) | - | 79.0 (I+A) | 1.2 (1064) | 4.8 (1064) | N/A | N/A | N/A | N/A |
| 7 | 4.3 (400) | 25.3 (400) | 60.0 (I+A) | N/A | N/A | N/A | N/A | N/A | N/A |
| 8 | 2.8 (393) | 19.6 (393) | 40.8 (I+A) | N/A | N/A | N/A | N/A | N/A | N/A |
| 9 | - | - | 36.8 (I+A) | N/A | N/A | N/A | N/A | N/A | N/A |
| 10 | 5.5 (60)^4^ | 16.4 (422) | N/A | N/A | N/A | N/A | N/A | N/A | N/A |

N/A not applicable; - not available

^1^ Baseline survey with age-stratified infection prevalence available

^2^ Baseline survey with overall community infection prevalence available

^3^ I: ivermectin, A: albendazole, D: diethylcarbamazine

^4^ expected community mf positive based on sub-sample as only those who were CFA positive were tested for mf

^5^average of previous four rounds

Table D. Model parameters

| Parameter | Definition (units) | Prior range | References |
| --- | --- | --- | --- |
|  |  |  |  |
| *λ* | Number of bites per vector (per month) | [5, 15] | [6-10] |
| *V/H* | Ratio of number of vectors to hosts | MBR^1^ / *λ* | data |
| *H_Lin_^2^* | Threshold value used in *h(a)* to adjust the age-dependent exposure rate (months) | [240, 360] | [6,8,11] |
| *A^2^* | Coefficient describing population age distribution in *π(a)* | data | data |
| *B^2^* | Coefficient describing population age distribution in *π(a)* | data | data |
| *ψ_1_* | Proportion of L3 leaving vector per bite | [0.1, 0.8] | [12] |
| *ψ_2_* | Larval establishment rate^3^ | [0.00003, 0.00364] | [6-8,13] |
| *c* | Strength of acquired immunity | [0.015, 0.025] | [6-8] |
| *I_C_* | Strength of immunosuppression^4^ | [0.5, 5.5] | [6-8] |
| *S_C_* | Slope of immunosuppression function^5^ (per worm/month) | [0.01, 0.20] | [6-8] |
| $\delta$ | Immunity waning rate (per month) | [0.001, 0.01] | [6-8] |
| *μ_W_* | Worm mortality rate (per month) | [0.008, 0.018] | [6-8,14-17] |
| *τ* | Pre-patency period (months) | [1, 9] | [18] |
| *k_0_* | Basic location parameter of negative binomial distribution used in *k* pertaining to worms | [0.000036, 0.000775] | [6-8,19,20] |
| *k_Lin_* | Linear rate of increase in *k* pertaining to worms | [0.00000024, 0.282] | [6-8,19,20] |
| *k_0A_* | Basic location parameter of negative binomial distribution used in *k* pertaining to antigen | [0.000036, 0.0015] | [6-8,19,20] |
| *k_LinA_* | Linear rate of increase in *k* pertaining to antigen | [0.00000024, 0.5640] | [6-8,19,20] |
| *s* | Proportion of female worms | 0.5 | - |
| *α* | Production rate of microfilariae per worm (per month) | [0.25, 1.5] | [6-8,12] |
| *γ* | Microfilariae mortality rate (per month) | [0.08, 0.12] | [6,8,12,16] |
| *α_2_* | Production rate of circulating filarial antigen (per month) | 0.5, 9.370 | [21] |
| *γ_2_* | Decay rate of circulating filarial antigen (per month) | 0.0125, 0.2 | [21] |
| *b* | Proportion of vectors which pick up infection when biting an infected host | [0.251, 0.485] | [6,8,22] |
| *κ* | Maximum level of L3 given mf density | [3, 5] | [6,8] |
| *r* | Gradient of mf uptake^6^ | [0.04, 0.25] | [6,8] |
| *σ* | Vector mortality rate (per month) | [1.5, 8.5] | [6,8,20] |
| *ω* | Worm killing efficacy of drug (instantaneous) | [0, 0.2] for IVM, [0.25, 0.45] for IVM+ALB, [0.45, 0.65] for DEC+ALB | [23] |
| *ε* | Microfilariae killing efficacy of drug (instantaneous) | Fixed at 0.99 for IVM and IVM+ALB, 0.95 for DEC+ALB | [23] |
| *δ_reduc_* | Reduction in the worm’s fecundity over a period of time p due to drug | Fixed at 0.99 for IVM and IVM+ALB, 0.95 for DEC+ALB | [23] |
| *p* | A time period during which the drug remains efficacious in reducing the fecundity of the surviving adult worms | Fixed at 9 for IVM and IVM+ALB, 6 for DEC+ALB | [23] |
| *C* | Percentage of the population administered the drug | data | data |
| *η_1_* | Proportion of vectors deterred by LLINs | Fixed at 0.2 | [21,24,25] |
| *η_2_* | Level of feeding inhibition by LLINs | Fixed at 0.9 | [21,24,25] |
| *η_3_* | Toxicity of LLINs | Fixed at 0.97 | [21,24,25] |
| *Λ* | Decay rate of LLIN efficacy (per year) | Fixed at 0.26 | [21,24,25] |

^1^Note MBR (monthly biting rate) serves as an input to initialize the model, measured as bites per person per month, the value of which may be obtained from entomological surveys conducted in study sites. In the absence of the observed MBR value, the model has been adapted to estimate it from the community-level mf prevalence data.

^2^The parameters *A*, *B*, and *H_Lin_* are estimated from national human demographic data or from the age-prevalence data.

^3^The proportion of L3-stage larvae infecting human hosts that survive to develop into adult worms [7].

^4^The facilitated establishment rate of adult worms due to parasite-induced immunosuppression in a heavily infected human host.

^5^The initial rate of increase by which the strength of immunosuppression is achieved as *W* increases from 0 [26].

^6^The gradient of mf uptake *r* is a measure of the initial increase in the infective L3 larvae uptake by vector as *M* increases from 0 [7,11].

Table E. Model functional forms

| Function | Functional form |
| --- | --- |
| $h(a)$: age-dependent exposure rate | $=\left\{\begin{matrix} \frac{a}{H_{Lin}}\text{ for }a<H_{Lin} \\ 1\text{ for }a\geq H_{Lin} \end{matrix}\right.$ |
| $k$: parasite aggregation | $=k_{0}+k_{Lin}\overline{M(a,t)}$ |
| $\zeta$: rate of pre-patent worm maturation | $=e^{-\mu\tau}$ |
| :worm mating probability | $=1-\left(1+\frac{W}{2k}\right)^{-(1+k)}$ |
| $\pi(a)$: population age distribution | $=Ae^{(-Ba)}$ |
| $f[M(a,t),k]$: Vector Mf uptake response |   for vectors with cibarial armature (i.e. Anopheles mosquitoes)  for vectors without cibarial armature (i.e. Culex mosquitoes) |
| $\Phi$: larval establishment rate | $=\lambda\frac{V}{H}h(a)\psi_{1}\psi_{2}$ |
| $F_{1}$ : human immunity to larval establishment | $=\frac{1}{1+cI(a,t)}$ |
| $F_{2}$ : human immunosuppression | $=\frac{1+I_{C}S_{C}W_{T}(a,t)}{1+S_{C}W_{T}(a,t)}$ |
| $F_{3}$: Mf production in the human host |  |
| $F_{4}$: L3 stage larval density in the vector | $=\frac{\lambda\kappa b\int\pi(a)(1-f[M(a,t),k])da}{\sigma+\lambda\psi_{1}}$ |

## Modelling mass drug administration and bednet interventions

Intervention by mass drug administration was modeled based on the assumptions that anti-filarial treatment with a combination drug regimen acts by killing certain fractions of the populations of adult worms and microfilariae instantly after the drug administration. These effects are incorporated into the basic model by calculating the population sizes of worms and microfilariae as follows:

$$\left.\begin{matrix} P(a,t+dt)=(1-\omega C)P(a,t) \\ W(a,t+dt)=(1-\omega C)W(a,t) \\ M(a,t+dt)=(1-\varepsilon C)M(a,t) \end{matrix}\right\} \text{ at time }t=T_{MDA_{i}}$$

where *dt* is a short time period since the *i*th MDA was administered. During this short time interval, a given proportion of adult worms and microfilariae are instantly removed. The parameters *ω* and *ε* are drug killing efficacy rates for the two life stages of the parasite while the parameter *C* represents the MDA coverage. Apart from instantaneous killing of microfilariae, the drug continues to kill the newly reproduced Mf by any surviving adult worms at a rate *δ_reduc_* for a period of time, *p*. We model this effect as follows:

The first MDA round was implemented in the model by affecting the population sizes of worms and microfilariae from the baseline estimates, and then the intervention is simulated forward in time for a number of years, with subsequent MDA rounds implemented annually.

In addition to MDA, we also modeled the added effect of long lasting insecticidal nets (LLINs) as described previously [21]. The impact of LLINs with three main actions against mosquito biting was modelled: 1) deterrence from entering the home (efficacy *η_1_*), 2) inhibition of their ability to feed on humans (efficacy *η_2_*), and 3) killing them (efficacy *η_3_*) [24,25]. To capture these effects, which decay over time as the larvicide efficacy declines exponentially at rate *Λ*, we adjust the term *V/H* to be appropriately modified according to the population coverage (*C_VC_*): $\frac{V}{H}\left(1-\eta_{1}\exp\left(-\Lambda t\right)C_{VC}\right)\left(1-\eta_{2}\exp\left(-\Lambda t\right)C_{VC}\right)\left(1-\eta_{3}\exp\left(-\Lambda t\right)C_{VC}\right)$

We assume that LLINs are replaced every three years.

Table F. 2003 mid-MDA survey age profile data for Seri, Gbuwhen, and Maiganga, Nigeria.

|  | Seri | | Gbuwhen | | Maiganga | |
| --- | --- | --- | --- | --- | --- | --- |
| Age group | mf prevalence (%) (no. sampled) | CFA prevalence (%) (no. sampled) | Mf prevalence (%)(no. sampled) | CFA prevalence (%)(no. sampled) | Mf prevalence (%)(no. sampled) | CFA prevalence (%)(no. sampled) |
| 0-9 | 1.1 (91) | 4.4 (91) | 1 (198) | 4.5 (198) | 0.6 (174) | 3.4 (177) |
| 10-19 | 7.2 (194) | 29.9 (194) | 1 (99) | 11.9 (101) | 4.5 (111) | 20.4 (113) |
| 20-29 | 14.7 (102) | 43.1 (102) | 7 (86) | 23.3 (86) | 10.1 (69) | 29 (69) |
| 30-39 | 15.4 (26) | 50 (26) | 7 (57) | 22.8 (57) | 4.1 (49) | 34.7 (49) |
| 40-49 | 19.2 (78) | 52.6 (78) | 7.4 (27) | 18.5 (27) | 15.4 (26) | 34.6 (26) |
| 50-59 | 14.3 (28) | 50 (28) | 25 (16) | 37.5 (16) | 10.5 (19) | 20 (20) |
| 60+ | 33.3 (9) | 44.4 (9) | 0 (8) | 50 (8) | 9.5 (21) | 33.3 (24) |


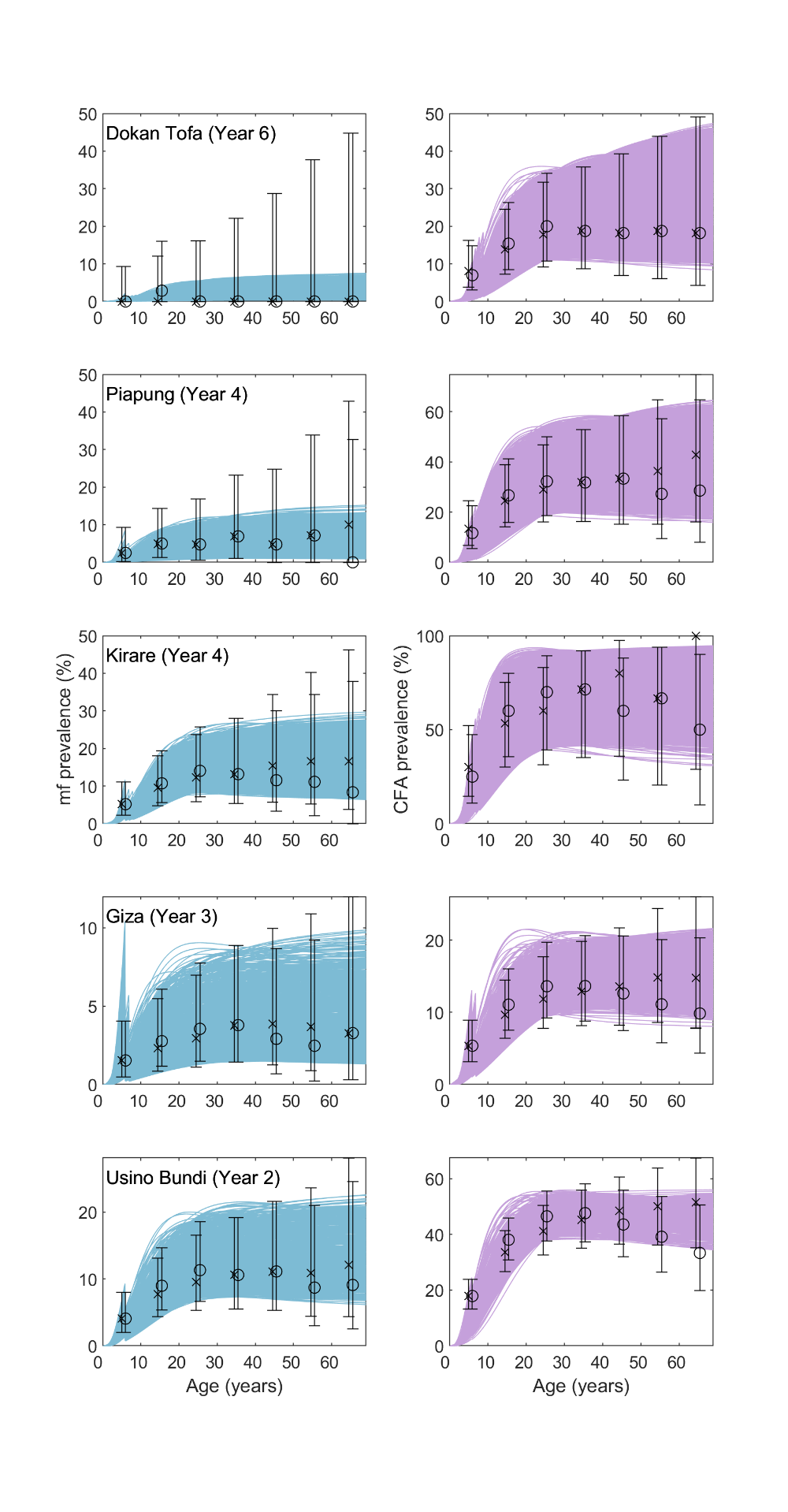


Fig A. Model fits to mid-MDA mf and CFA survey data. Each row of plots give the mf (left, blue curves) and CFA (right, purple curves) age prevalence for the given site at the indicated survey year. The curves represent the model-predicted infection by age compared to the derived plateau (crosses) and convex (circles) age infection profiles. The age profiles and their 95% confidence intervals were derived from the observed overall community mf and CFA infection in each site as described in [27] and the main text methods section “Pass/fail model selection based on mid-MDA data”. Overall Monte-Carlo p-values > 0.9 for each data set indicating no significant difference between model predictions and the derived data.


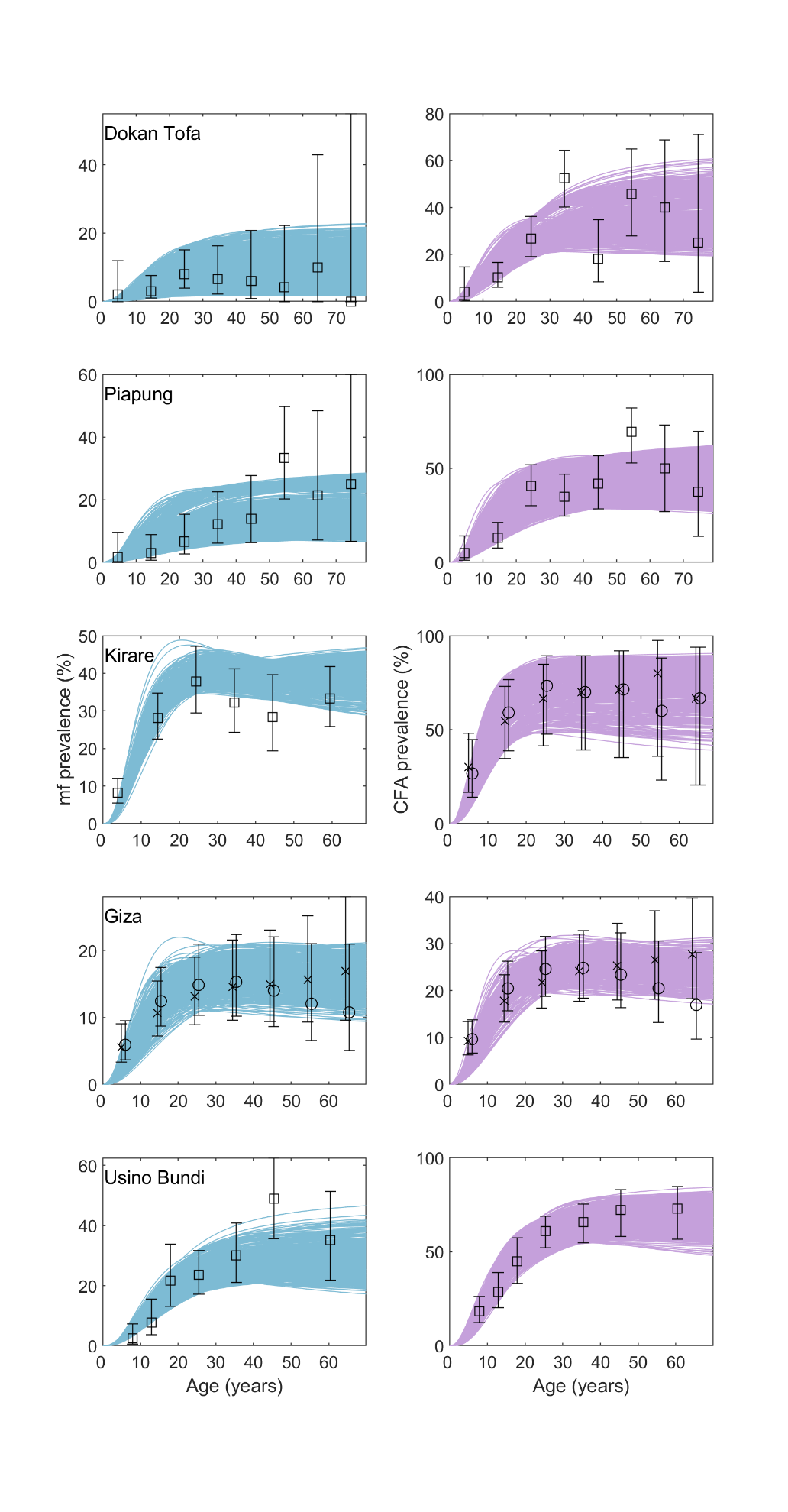


Fig B. Direct model fits to observed baseline mf and CFA infection prevalence data. Each row of plots give the mf (left) and CFA (right) age prevalence for the given site at baseline. The gray curves represent the model-predicted infection by age compared to the observed (black) or derived (plateau as red points and convex as blue points) age infection profiles. Overall Monte Carlo p-values > 0.9 for each site indicate no significant difference between model predictions and observed data.


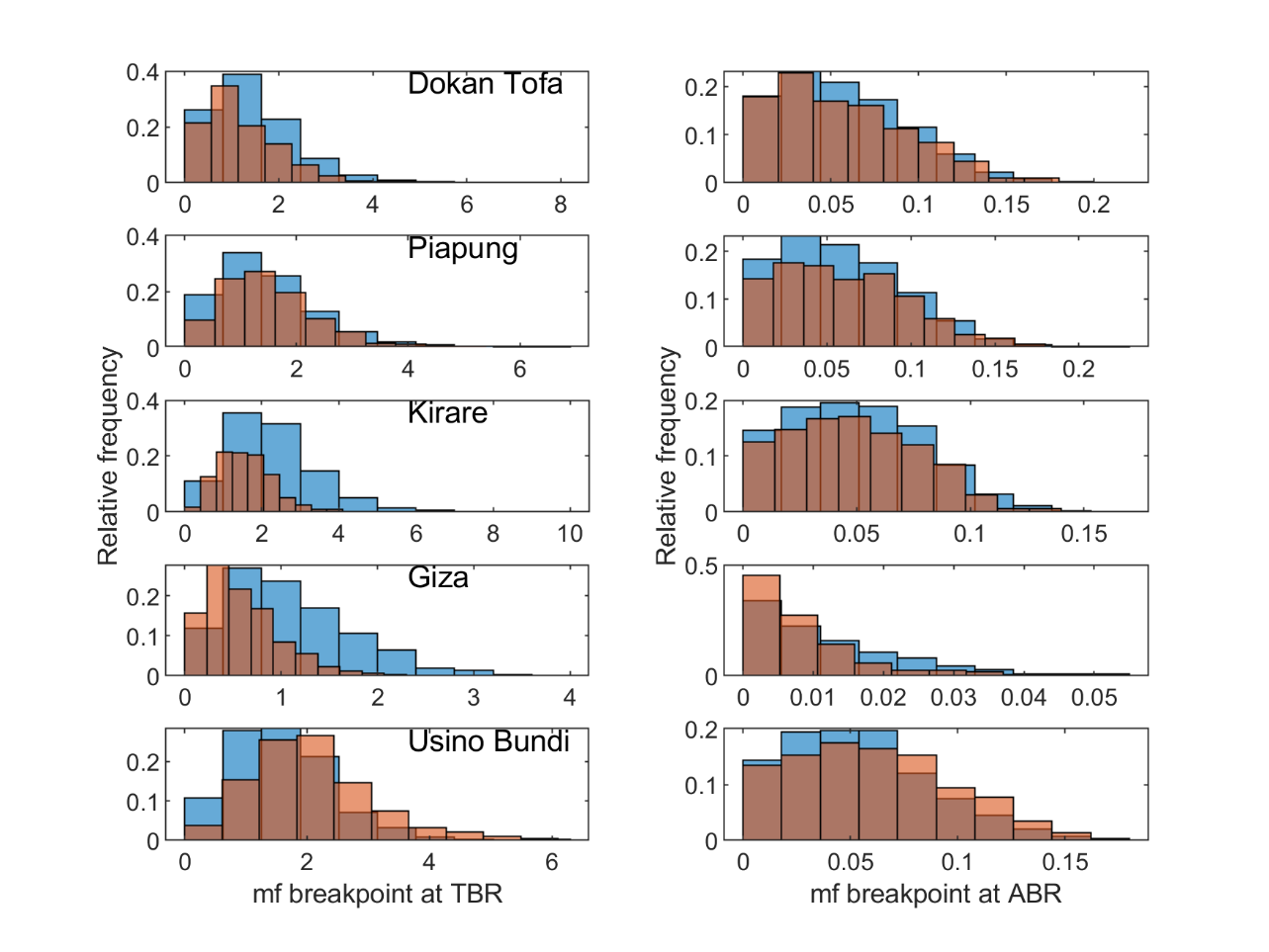


Fig C. Distributions of mf breakpoints predicted by the hindcasted models (blue) compared to the predictions of the models fitted directly to baseline (orange). Each row gives the comparison for the mf breakpoints at TBR and ABR for the indicated site.

Table G. Kruskal Wallis tests comparing the breakpoint distributions from direct model fits and hindcasts.

| Village | Kruskal Wallis p-value | |
| --- | --- | --- |
|  | Breakpoints at ABR | Breakpoints at TBR |
| Dokan Tofa | 0.35 | 1.82 e-13 |
| Piapung | 0.52 | 0.09 |
| Kirare | 0.43 | 3.34 e-47 |
| Giza | 9.22 e-13 | 5.12 e-54 |
| Usino Bundi | 2.17 e-4 | 2.75 e-21 |

Table H. Comparison of predicted timelines to reach the 1% mf threshold from models directly fitted to baseline data versus hindcasted models.

| Village | Median (95% CI) number of rounds of MDA required | |
| --- | --- | --- |
|  | Direct fits to baseline | Hindcasts |
| Dokan Tofa | 3 (2-6) | 5 (2-8) |
| Piapung | 5 (3-15) | 5 (3-8) |
| Kirare | 10 (8-12) | 11 (10-14) |
| Giza | 4 (3-50) | 4 (3-50) |
| Usino Bundi | 5 (4-7) | 5 (4-7) |


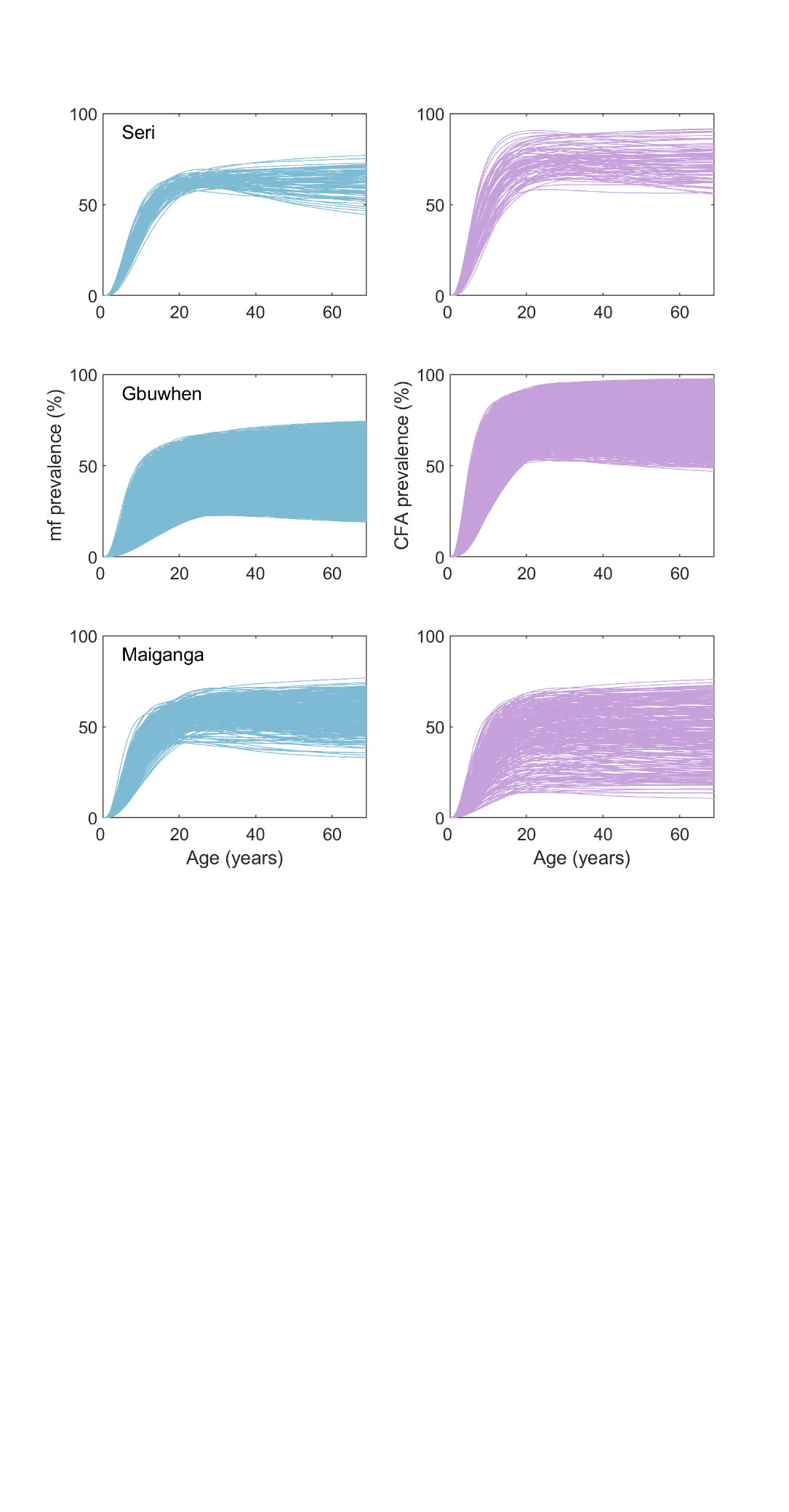


Fig D. Model hindcasts of baseline mf and CFA prevalence in three sites without baseline data. Each row of plots give the model-predicted mf (left, blue curves) and CFA (right, purple curves) baseline age prevalence predictions for the given site.

References

1. Simonsen PE, Pedersen EM, Rwegoshora RT, Malecela MN, Derua YA, Magesa SM. Lymphatic filariasis control in Tanzania: effect of repeated mass drug administration with ivermectin and albendazole on infection and transmission. PLoS Negl Trop Dis. 2010;4: e696.

2. Simonsen PE, Derua YA, Kisinza WN, Magesa SM, Malecela MN, Pedersen EM. Lymphatic filariasis control in Tanzania: effect of six rounds of mass drug administration with ivermectin and albendazole on infection and transmission. BMC Infect Dis. 2013;13: 335.

3. Simonsen PE, Derua YA, Magesa SM, Pedersen EM, Stensgaard A, Malecela MN, et al. Lymphatic filariasis control in Tanga Region, Tanzania: status after eight rounds of mass drug administration. Parasit Vectors. 2014;7: 1.

4. Ramzy RM, El Setouhy M, Helmy H, Ahmed ES, Elaziz KMA, Farid HA, et al. Effect of yearly mass drug administration with diethylcarbamazine and albendazole on bancroftian filariasis in Egypt: a comprehensive assessment. Lancet. 2006;367: 992-999.

5. Weil GJ, Kastens W, Susapu M, Laney SJ, Williams SA, King CL, et al. The impact of repeated rounds of mass drug administration with diethylcarbamazine plus albendazole on bancroftian filariasis in Papua New Guinea. PLoS Negl Trop Dis. 2008;2: e344.

6. Gambhir M, Michael E. Complex ecological dynamics and eradicability of the vector borne macroparasitic disease, lymphatic filariasis. PLoS One. 2008;3: e2874. doi: 10.1371/journal.pone.0002874.

7. Gambhir M, Bockarie M, Tisch D, Kazura J, Remais J, Spear R, et al. Geographic and ecologic heterogeneity in elimination thresholds for the major vector-borne helminthic disease, lymphatic filariasis. BMC Biol. 2010;8: 8-22.

8. Singh BK, Bockarie MJ, Gambhir M, Siba PM, Tisch DJ, Kazura J, et al. Sequential modelling of the effects of mass drug treatments on anopheline-mediated lymphatic filariasis infection in Papua New Guinea. PLoS One. 2013;8: e67004.

9. Rajagopalan PK. Population dynamics of culex pipiens fatigans, the filariasis vector, in pondicherry: influence of climate and environment. Proc Indian Natl Sci Acad. 1980;46: 745-752.

10. Subramanian S, Manoharan A, Ramaiah KD, Das PK. Rates of Acquisition and Loss of Wuchereria Bancrofti Infection in Culex Quinquefasciatus. Am J Trop Med Hyg. 1994;51: 244-249. doi: 10.4269/ajtmh.1994.51.244.

11. Norman RA, Chan M, Srividya A, Pani SP, Ramaiah KD, Vanamail P, et al. EPIFIL: the development of an age-structured model for describing the transmission dynamics and control of lymphatic filariasis. Epidemiol Infect. 2000;124: 529-541.

12. Hairston NG, de Meillon B. On the Inefficiency of Transmission of Wuchereria bancrofti from Mosquito to Human Host. Bull World Health Organ. 1968;38: 935-941.

13. Ho BC, Ewert A. Experimental transmission of filarial larvae in relation to feeding behaviour of the mosquito vectors. Trans R Soc Trop Med Hyg. 1967;61: 663-666. doi: 10.1016/0035-9203(67)90130-7.

14. Vanamail P, Subramanian S, Das PK, Pani SP, Rajagopalan PK. Estimation of fecundic life span of Wuchereria bancrofti from longitudinal study of human infection in an endemic area of Pondicherry (south India). Indian J Med Res. 1990;91: 293-297.

15. Evans DB, Gelband H, Vlassoff C. Social and economic factors and the control of lymphatic filariasis: A review. Acta Trop. 1993;53: 1-26. doi: 10.1016/0001-706X(93)90002-S.

16. Ottesen EA, Ramachandran CP. Lymphatic filariasis infection and disease: Control strategies. Parasitol Today. 1995;11: 129-131. doi: 10.1016/0169-4758(95)80128-6.

17. Vanamail P, Ramaiah KD, Pani SP, Das PK, Grenfell BT, Bundy DaP. Estimation of the fecund life span of Wuchereria bancrofti in an endemic area. Trans R Soc Trop Med Hyg. 1996;90: 119-121. doi: 10.1016/S0035-9203(96)90106-6.

18. Scott AL. Lymphatic-dwelling Filariae. In: Anonymous Lymphatic Filariasis. London, UK: Imperial College Press; 2000. pp. 5-39.

19. Subramanian S, Pani SP, Das PK, Rajagopalan PK. Bancroftian filariasis in Pondicherry, South India: 2. Epidemiological evaluation of the effect of vector control. Epidemiol Infect. 1989;103: 693-702.

20. Das PK, Manoharan A, Subramanian S, Ramaiah KD, Pani SP, Rajavel AR, et al. Bancroftian filariasis in Pondicherry, south India–epidemiological impact of recovery of the vector population. Epidemiol Infect. 1992;108: 483-493.

21. Singh BK, Michael E. Bayesian calibration of simulation models for supporting management of the elimination of the macroparasitic disease, lymphatic filariasis. Parasit Vectors. 2015;8: 522.

22. Subramanian S, Krishnamoorthy K, Ramaiah KD, Habbema JDF, Das PK, Plaisier AP. The relationship between microfilarial load in the human host and uptake and development of Wuchereria bancrofti microfilariae by Culex quinquefasciatus: a study under natural conditions. Parasitology. 1998;116: 243-255.

23. Michael E, Malecela-Lazaro MN, Simonsen PE, Pedersen EM, Barker G, Kumar A, et al. Mathematical modelling and the control of lymphatic filariasis. Lancet Infect Dis. 2004;4: 223-234.

24. Okumu FO, Moore SJ. Combining indoor residual spraying and insecticide-treated nets for malaria control in Africa: a review of possible outcomes and an outline of suggestions for the future. Malar J. 2011;10: 208.

25. Griffin JT, Hollingsworth TD, Okell LC, Churcher TS, White M, Hinsley W, et al. Reducing Plasmodium falciparum malaria transmission in Africa: a model-based evaluation of intervention strategies. PLoS Med. 2010;7: e1000324.

26. Duerr H, Dietz K, Eichner M. Determinants of the eradicability of filarial infections: a conceptual approach. Trends Parasitol. 2005;21: 88-96.

27. Smith ME, Singh BK, Michael E. Assessing endgame strategies for the elimination of lymphatic filariasis: A model-based evaluation of the impact of DEC-medicated salt. Sci Rep. 2017;7: 7386-12.
