# Supplementary material for: Predicting lymphatic filariasis elimination in data-limited settings: A reconstructive computational framework for combining data generation and model discovery
Source: PLoS Comput Biol. 2020 Jul 21;16(7):e1007506. doi: 10.1371/journal.pcbi.1007506 (PMC7394457; doi:10.1371/journal.pcbi.1007506)
Supplement: Attachment — Submitted filename: ReviewerResponseLetter_27 Mar2020.docx [file pcbi.1007506.s002.docx]

Dear Editors,

Thank you for inviting us to resubmit a revised version of our manuscript taking substantive account of reviewers’ comments. We thank both the reviewers for their appraisal of the manuscript and appreciate their input. We are pleased to hear that they found the work of value. We have addressed their concerns below in a point-by-point response and have made the corresponding changes to the revised manuscript submitted with this letter. Note all references to line and page numbers in our response allude to the tracked version of our revised ms. We hope that these revisions will be satisfactory and will now allow our work to be published.

**Reviewer comments:**

**Reviewer #1:**

The authors have constructed a detailed mathematical analysis of LF transmission in selected sites, engaging in a type of computational exploration which is billed as 'model discovery'. This is a well-established group in the field of LF modeling and have related their work to specific policy questions.

The heart of the presented novelty seems to be Figure 1, where we are told "site specific models" are identified. What does this mean? Does it mean the choice of appropriate parameters based on Table 4? Or does it mean statistical model selection? So little detail is provided that the claims made cannot be evaluated, let alone reproduced.

We thank the reviewer for this feedback, and apologize for any confusion caused in describing our method. Figure 1 is meant to be read in conjunction with the overview of our method given in the section “Computational system for data and model discovery” (p. 12) and the specific sub-sections on “Mathematical model of LF Transmission”, and model initialization and calibration respectively. Please note the final sentence of this section which points the reader to the subsequent and detailed methods sections (mathematical model, calibration procedures and other relevant calculation methods) that underpin our method (lines 218-219). Nonetheless we have made edits to explicate what exactly we mean by identification of “site-specific” models (lines 206-212), plus added the core model equations to the main text (under the mathematical model section to furnish greater detail).

Selected rather minor comments:
Line 24. "Increasing recognition of the importance of mathematical models" is a dubious claim, as is the remainder of the sentence regarding the plausible-sounding idea that "useful" models "must accurately reflect" processes "pertaining to local infection dynamics". What does _accurately_ really mean in this context? The authors go on to say the models "must be identified", and that is unclear also. Does this refer to statistical identifiability?

We have made edits to the beginning of the abstract to use less controversial language. There is no room in the abstract to discuss fully the reviewer’s questions about terminology, but we have amended wording where possible to take account of the reviewer’s comments. Note points made in the abstract are further expanded upon in the opening paragraphs of the introduction.

Line 39. Claim that the model has provided "critical" information; this is tendentious; recommend omit.

We have changed our choice in wording here.

Line 56. "knowledge discovery system'"; recommend omit. What knowledge? Are they talking about baseline values of prevalence?

We have modified the word choice here as well.

**Reviewer #2:**

General comments:
The paper presents interesting and useful methods and results that are very relevant to the current landscape of NTD elimination. However, the manuscript is written in a very dense and difficult to digest style. In a number of instances the reader is expected to follow up on multiple references at a time to disentangle single pieces of information vital to the reproducibility of the work. Having followed up on a number of these references it is not always clear what the authors are referring to, making the methods difficult to follow and substantiate. The sentence structure is also often long-winded and over-punctuated, with many sentences stretching over 5 or more lines of text.

The entire manuscript would benefit from rewording to include shorter and more digestible sentences. In addition, the authors should ensure clearer description of the modelling and statistical processes deployed, rather than relying on excessive referencing. Some of these can be including in supplementary but there are a number that would benefit from being included in the main text. This will ensure ease of understanding and reproducibility of the work described.

We thank the reviewer for the feedback on the value of this work and the suggestion to review the paper in full for clarity and readability. We have made changes throughout the manuscript and hope that its accessibility has improved.

Specific comments:

Abstract: Excessively long sentences (some up to 5 lines long) make it difficult to read. Consider revising.

We thank the reviewer for this suggestion, and we have revised the abstract to make it more readable.

49-51 “For many neglected…” Sentence structure is muddled - please revise.

We have revised this sentence accordingly.

89-92: Consider revising sentence, ideally into two shorter sentences

We have revised this sentence to reduce the number of clauses.

96-98: Repetition of “data” and general sentence structure/length.

We have revised this sentence.

206-208: This doesn’t currently make sense when read through - what does “or any other” refer to here? Please reword.

We have revised this sentence.

210: Why are there so many references here? To allow ease of reproducibility you should provide one or two of the most relevant and specific references for the methods being employed. Using a clump of 6 references when describing a single method is inappropriate.

We included a complete list of references in this overview section to demonstrate that the method has been well-established and used in our previous work. In the detailed Methods section that follows, (see line 252-253) the reader is referred to only two papers.

Line 245: How were parameter vectors selected to ensure community mf prevalence distribution was uniform? How did you select/reject samples?

We first randomly sampled N = 200,000 parameter vectors from the assigned parameter priors. We then simulated the endemic equilibrium given each parameter vector and calculated the predicted mf prevalence at baseline. Those parameter vectors whose outputs produced mf prevalence values between 5-50% were accepted while all others are rejected. To ensure that a uniform posterior distribution of prevalences was generated, we used the histogram function in Matlab to count the frequency of model predictions falling into 1% mf prevalence “bins” (ex. 20-21% mf, 21-22% mf, 22-23% mf, etc.). The accepted parameter vectors were then further pruned such that the same number of parameter vectors were accepted in each bin. This sampling and acceptance/rejection procedure was repeated until the total number of accepted parameter vectors was equal to 200,000. This is summarized under the section, “Initializing the model” (p. 14).

Line 252: Please expand on what this “pre-defined criteria of fit” is

The pass/fail criteria used is described in the subsequent sentences. We have added a phrase to this sentence to make the connection clearer (lines 251-254).

Line 256: Does “single mid-MDA survey” mean a single time point? Why was only a single time point used?

Yes, this refers to a single time point. A single survey was used primarily to reflect the typical data availability in the field. The WHO requires only baseline and one survey after 5 rounds of MDA with an optional survey after 3 years of MDA, so it is atypical to have many surveys to use for model fitting. The selection of the survey year for each site is described in the paragraph beginning on line 261. We have also published complementary research in which we investigated the potential benefits of fitting models to a longer series of data. In these work, we find that the parameter values are relatively stable during the intervention period and that there may not be significant changes if we fit to multiple post-intervention data points. We explore this topic in the discussion (see paragraph starting on line 497) and in the papers referenced below.

Singh BK, Bockarie MJ, Gambhir M, Siba PM, Tisch DJ, Kazura J, et al. Sequential modelling of the effects of mass drug treatments on anopheline-mediated lymphatic filariasis infection in Papua New Guinea. PLoS One. 2013;8: e67004.

Michael E, Sharma S, Smith ME, Touloupou P, Giardina F, Prada JM, et al. Quantifying the value of surveillance data for improving model predictions of lymphatic filariasis elimination. PLoS Negl Trop Dis. 2018;12: e0006674

Testing sites (Dokan Tofa, Piapung, Kirare, Giza and Usino Bundi) have no age-stratified mid-MDA data, the age profile has to be derived, so the model is essentially different in these sites than in Seri, Gbuwhen and Maiganga. Make this clearer, how does this impact validation?

The reviewer is correct, the quality of the mid-MDA data for model fitting differs as described in the paragraph starting on line 261. Calibration (mid-MDA) data for Seri, Gbuwhen, and Maiganga are age-stratified while the mid-MDA data for the other five sites are not. We therefore derived age-profiles from the overall community infection prevalence using an approach developed in previous work (ref 40). This approach generates likely age profiles that depend on the overall community prevalence and the population age structure of the country. We use an ensemble of two likely infection patterns over age (plateau - infection prevalence stays constant among older age groups, and convex - infection prevalence peaks at a certain age group and then declines in older age groups) to capture a range of possible patterns that maintain the observed overall prevalence in these sites. This introduces some level of uncertainty to the modelling process, so we expect that the ensemble of models in these sites would likely show more variation than if the age-stratified data was directly observed as for Seri, Gbuwhen, and Maiganga. However, this does not impact the goodness of fit significantly as shown by Monte Carlo p-values > 0.9 indicating no significant difference between model hindcasts and observed baseline data. In responding to this question, we made some edits to better clarify the availability of age-stratified data for model calibration and validation (see main text lines 263-267, and 197 (table 1 footnote) and footnotes in S1 Table 2 and S1 Table 3). We also added some text to the results section (line 357-361) to address this question. We thank the reviewer for raising this question for clarity.

273-281: The pruning method described here doesn’t appear to align with any described in the provided reference [47]. I may have missed it (feel free to point it out if so) but if not then please provide a suitable reference to confirm legitimacy of these pruning method.

Ref 47 was given to highlight that pruning is a general principle used for addressing uncertainty with ensemble predictions. Our approach described from line 273 was formulated to discard extreme predictions from the our ensemble.

297-299: How was the reduction in biting rate calculated? What is the functional relationship between ITN and LLIN coverage/efficacy and biting rate? You would expect ITN and LLIN usage to reduce the biting rate, but also reduce the prevalence of infection in the mosquito population, which does not appear to be considered here. The effect is commonly considered to be quadratic (repelling/preventing feeding slows disease acquisition and transmission).

We thank the reviewer for bringing this missing information to our attention. We have added a description of our vector control equations to the supplementary information fully now.

312-313: Waning effect of bednets?

Our model assumes bednets are replaced periodically. This is now presented in full in the supplementary information as above.

Recrudescence is not a suitable word as used in this manuscript. In a medical sense, recrudescence is the return of detectable symptoms in a patient whose blood stream infection has previously been at such a low level as not to be clinically demonstrable or cause symptoms, it does not refer to the re-establishment of disease at a population level. Resurgence would be a more suitable word.

We have used the suggested word choice in the revised ms, but note the use of recrudescence is common place in infectious disease modelling to denote reinfection following control.

314-319: How are marginally positive and negative slopes dealt with that could simply represent a maintenance of low-levels of infection rather than a trend towards elimination or resurgence?

In this analysis, we have assumed that if there is not a clear resurgence after long time periods (10 years), then we can conclude the infection prevalence has been reduced to unsustainable levels and will decline in the long term. Our analysis to be published elsewhere has indicated that waiting this long to determine trends toward elimination or resurgence is sufficient to classify these transmission outcomes.

323: Reference for Bayesian melding framework should be repeated here.

Thank you for the suggestion, we have added the reference.

324-328: Is this supposed to describe the Bayesian melding framework? It sounds like basic approximate bayesian computation. Also the “binary criteria of fit” is not described anywhere in the manuscript. It is mentioned, with reference, in line 252 but not elaborated on what this actually means. This is a vital part of the methodology for reproducibility, please specify explicitly within the manuscript.

Yes, we are using a variation of Bayesian Melding originally proposed by Spear and Hubbard but we agree that it may also fit the description of ABC methods.

Spear RC, Hubbard A. Parameter estimation and site-specific calibration of disease transmission models. In: Michael E, Spear RC, editors. Modelling Parasite Transmission and Control. New York: Springer; 2010. pp. 99-111.

Please see lines 256-259 for a description of the criteria for acceptance/rejection.

332: “fit” should be “fitted”

Thank you, we have corrected this.

Figure 1: It’s very hard to see the crosses and circles, with their respective error bars, where they are overlaid. Also the area covered by the model-derived curves is most of the space in a number of plots (e.g. Dokan Tofa CFA). This doesn’t represent an accurate estimate of baseline prevalence, simply that the observed baseline prevalence lies within the very wide range of possibilities predicted by the model. An estimated baseline mf prevalence of between 0 and up to 50% in some age categories (Dokan Tofa, Giza, Piapung) isn’t particularly informative.

We have hopefully addressed this better now (new Fig 2) by jittering the X-axis for sites where age-derived data were used for model-fitting. We did statistically evaluate the information loss by using this method, wherein in Table 2 we show that information is indeed lost by hindcasting to baseline as opposed to fitting directly to baseline data. However, we noted (lines 358- 362) that despite this, our reconstructed models from such data are still able to predict historic prevalence levels sufficiently enough to be valuable for control program planning.

Table 3: Is this baseline prevalence? Specify in title.

Thank you for catching this, we have added detail to the title to indicate the data is from 2003 after MDA had already occurred for onchocerciasis.

Table 4: Is ABR here calculated in the complete absence of vector control or is this assuming the low coverage of vector control assumed in the model? Also a number of these (Dolan Tofa, Piapung, Kirare, Usino Bundi, Seri, and Gbuwhen) are within reasonable error of 0.01 mf (1% prevalence), or at least could not be called “markedly lower” as described in line 414. This is reflected by the very low (or zero) resurgence probabilities presented for these settings in Table 5. The highest resurgence probability is seen in Giza, where no bednets are present, which implies to me that the ABR presented in Table 4 may include local bednet usage. This is misleading as it suggests that transmission is naturally higher in Giza, but may mainly be due to a lack of bednet usage.

Yes, the breakpoints at ABR are calculated in the absence of vector control. In sites where bednets are not used (ex. Giza), we use the breakpoint at ABR provided in Table 4 as the target threshold to reach before stopping MDA treatments. In sites where VC is used (all others), we use the breakpoints at TBR as the target threshold.

The breakpoints in Table 4 are given as prevalence – for example, Giza’s breakpoint at ABR is 0.001% which is two orders of magnitude smaller than the WHO threshold of 1%. This is why we have commented that thresholds in some settings could be “markedly lower”. The thresholds at TBR are closer to the 1% threshold, but they are consistently estimated to be lower than 1% and assume that VC has been implemented which is a stronger intervention strategy than required by the WHO.

The reviewer is correct that the high resurgence probability seen in Giza is likely due to a lack of bednets, and we have discussed this conclusion regarding the apparent protective effect of VC in lines 450-455 in the results and lines 570-587 in the discussion.

No rigorous statistical tests of goodness of fit are used in the methods, please justify.

We have added goodness of fit statistics to the supplementary information and in the text here relevant using the Monte-Carlo p-value goodness of fit approach as detailed in reference 21. A sentence is also added regarding the use of this method for assessing model fits in lines 259-260.

Line 498: This is the range of point estimates with no uncertainty, could be misleading. Including uncertainty these are actually 16-52% mf and 18-91% CFA

We have added uncertainty to our description in the text (lines 396-399).

**Have all data underlying the figures and results presented in the manuscript been provided?**
Large-scale datasets should be made available via a public repository as described in the *PLOS Computational Biology* [data availability policy](http://journals.plos.org/ploscompbiol/s/data-availability), and numerical data that underlies graphs or summary statistics should be provided in spreadsheet form as supporting information.

Reviewer #1: No: I can't tell where the data shown in Supplement Figure 1 are.

We have added detail to the last sentence in the figure caption which now reads “The age profiles and their 95% confidence intervals were derived from the observed overall community mf and CFA infection in each site as described in Smith et al 2017 and the main text methods section “Pass/fail model selection based on mid-MDA data”.

Reviewer #2: Yes
